# Impact of an abdominal belt on breathing patterns and scan efficiency in whole-heart coronary magnetic resonance angiography: comparison between the UK and Japan

**DOI:** 10.1186/1532-429X-13-71

**Published:** 2011-11-17

**Authors:** Masaki Ishida, Andreas Schuster, Shinichi Takase, Geraint Morton, Amedeo Chiribiri, Boris Bigalke, Tobias Schaeffter, Hajime Sakuma, Eike Nagel

**Affiliations:** 1 King's College London British Heart Foundation (BHF) Centre of Excellence; National Institute of Health Research (NIHR) Biomedical Research Centre at Guy's and St. Thomas' NHS Foundation Trust; Wellcome Trust and Engineering and Physical Sciences Research Council (EPSRC) Medical Engineering Centre; Division of Imaging Sciences and Biomedical Engineering, The Rayne Institute, St. Thomas' Hospital, London, UK; 2 Department of Radiology, Mie University Hospital, Tsu, Japan

## Abstract

**Background:**

Long acquisition times and complex breathing motion patterns lead to suboptimal image quality in whole heart coronary magnetic resonance angiography (WHCMRA). To overcome this problem, an abdominal belt (BELT) has been suggested by a Japanese group. However, its applicability in a Western population has not been previously demonstrated. The purpose of this study wa**s **to investigate 1) how the application of a BELT alters breathing patterns during MR scanning and 2) whether the BELT has a similar impact on breathing patterns in UK and Japanese patient populations.

**Methods:**

30 patients (15 in the UK and 15 in Japan) were studied at 1.5 Tesla (Achieva, Philips Healthcare). Real time navigator positioned through the right diaphragm in cranio-caudal direction was evaluated. Measurements were performed in the supine position with free breathing for one minute before and after a tight-fitting BELT was positioned around the patient's abdomen. End expiratory position (EEP), end inspiratory position (EIP), end expiratory duration (EED) for the right diaphragm and respiratory rate (RR) were obtained. Scan efficiency (SE) was calculated as follows; SE = [the duration within 5 mm gating window per minutes]/[RR interval]/[heart rate].

**Results:**

Height and weight of UK patients were significantly larger than in the Japanese population (171.2 ± 10.8 cm vs 160.8 ± 8.5 cm, p = 0.007; 80.5 ± 22.5 kg vs 59.9 ± 7.7 kg, p = 0.004). After fitting the BELT, EEP-EIP decreased (all patients, 14.9 ± 6.2 mm to 9.4 ± 3.8 mm, p < 0.001; UK patients, 15.9 ± 6.0 mm to 9.7 ± 3.1 mm, p = 0.001; Japanese patients, 14.0 ± 6.4 mm to 9.1 ± 4.6 mm, p = 0.001), RR increased (all patients, 10.0 ± 3.1 min^-1 ^to 11.2 ± 3.0 min^-1^, p = 0.003; UK patients, 9.5 ± 2.8 min^-1 ^to 10.7 ± 2.8 min^-1^, p = 0.038; Japanese patients, 10.4 ± 3.5 min^-1 ^to 11.8 ± 3.1 min^-1^, p = 0.036), and calculated scan efficiency increased (all patients, 45.3 ± 11.4% to 58.6 ± 17.0%, p < 0.001; UK patients, 44.2 ± 10.8% to 55.7 ± 16.7%, p = 0.004; Japanese patients, 46.3 ± 32.2% to 61.0 ± 17.6%, p = 0.001). No significant differences were found between UK and Japanese patients before and after administration of the BELT.

**Conclusion:**

Using a BELT significantly increases whole-heart coronary MR angiography scan efficiency in both UK and Japanese patients.

## Background

Coronary artery disease (CAD) is one of the most frequent causes of death in many industrial countries [1]. There is a compelling requirement for noninvasive testing without ionizing radiation that can reliably detect CAD. There have been considerable technical advances in the field of coronary MR angiography (MRA) [2-5], and whole-heart coronary MRA can now be used to visualize the coronary arteries without administration of MR contrast medium [6]. Previous studies showed that whole-heart coronary MRA has a moderate sensitivity (78-82%) and a high specificity (91-98%) for detecting luminal narrowing of ≥50% in the coronary arteries on X-ray angiography [7-10]. In particular the value of whole-heart coronary MRA in patients with a low pre-test likelihood is similar to computed tomography coronary angiography [11]. The results from these previous studies have indicated the potential value of whole-heart coronary MRA for ruling out CAD.

Whole-heart coronary MRA is acquired during free breathing with a respiratory gating method using navigator echo techniques which track the motion of right hemi-diaphragmatic dome [10,12]. However, the major drawback of this free breathing technique is the relatively long acquisition time ranging from 10 to 20 minutes [7-10]. This is a result of the requirement to synchronize imaging with the cardiac and the breathing cycle and complex motion patterns. The long imaging time required for this approach results in an increased susceptibility to motion problems such as drift of the diaphragm position or heart rate variations which can lead to suboptimal image quality and unsuccessful scanning. Consequently, the success rate of whole-heart coronary MRA still remains in the range of 86% to 92% [7-10].

To overcome this problem, several approaches have been introduced such as drift correction to increase scan efficiency [12] and the multi-channel cardiac coils enabling higher sensitivity encoding acceleration factors [13]. In addition to these methods, the abdominal belt (BELT), which can suppress the abdominal breathing motion and thus improve whole-heart coronary MRA image quality, has been suggested by a Japanese group [10]. The BELT technique is widely used to acquire whole-heart coronary MR angiography in Japan because this technique is empirically known to improve the image quality of whole heart coronary MR angiography. It is not known whether this also applies to a Western patient population where patient size and body habitus differ largely from the Japanese population [14]. We sought to compare the effects of this relatively unobtrusive intervention on whole-heart coronary MR angiography image quality in both Western and Japanese patient populations. However, the mechanism by which this approach improves whole-heart coronary MRA image quality remains unclear. More importantly, its applicability in a Western population has not been demonstrated.

The purpose of this study is 1) to investigate how the BELT improves image quality of whole-heart coronary MRA and 2) whether the BELT has similar impact on breathing patterns in the UK and Japanese patient populations.

## Methods

### Patients

We studied 30 patients (15 patients each in the UK and Japan) randomly selected from the patients who were referred for a routine clinical cardiovascular MR scan. Exclusion criteria included patients with general contraindications to MRI (e.g., pacemakers, claustrophobia), abdominal aortic aneurysm, pregnancy, severe pulmonary disease, heart failure (NYHA class III-IV) and any abdominal or thoracic pain. Patients' characteristics are shown in table 1. The local ethics committee of both institutes approved the study, and all patients gave written informed consent to participate.

**Table 1:** Patient characteristics

	Total (n = 30)	UK (n = 15)	Japan (n = 15)	p
Age (years)	59.5 ± 14.0	50.7 ± 11.9	68.2 ± 10.0	< 0.001

Male/female	19/11	7/8	12/3	

Height (cm)	166.0 ± 10.9	171.2 ± 10.8	160.8 ± 8.5	0.007

Weight (kg)	70.2 ± 19.6	80.5 ± 22.5	59.9 ± 7.7	0.004

BSA	1.79 ± 0.28	1.94 ± 0.31	1.64 ± 0.14	0.003

Abdominal circumference (cm)	92.0 ± 15.8	99.1 ± 19.2	85 ± 6.7	0.016

Heart rate (bpm)	71 ± 12	69 ± 9	73 ± 13	0.287

### Acquisition of MRI data

**Figure 1:**
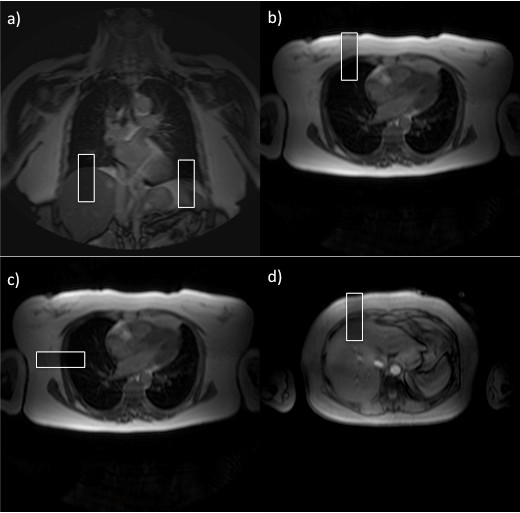
**Navigation position. **a) Navigator right and left diaphragm CC, b) navigator right thoracic wall AP, c) navigator right thoracic wall LR and d) navigator upper abdominal wall AP.

**Figure 2:**
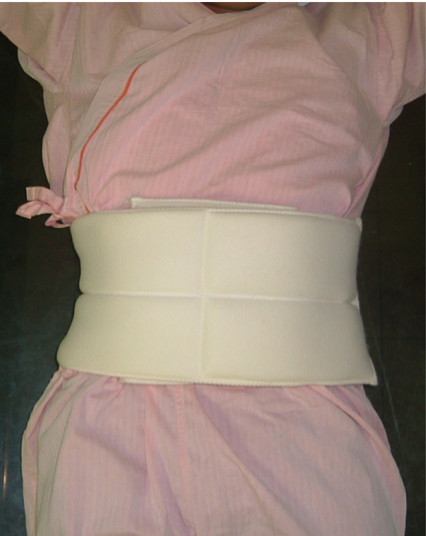
**The 20-cm-wide BELT**. The 20-cm-wide BELT was wrapped tightly around the patient's abdomen during end expiration to suppress diaphragmtic motion.

The MR examination was performed on a commercial 1.5 Tesla MRI unit (Achieva, Philips Healthcare, Best, The Netherlands) equipped with cardiac software (release 2.53) and a commercial gradient system (33 mTm peak on axis, 80 mT/m/ms slew rate). At the end of the clinical routine cardiac MR scan, the navigator acquisitions were performed [15]. Each navigator beam consisted of a cylindrical 2D spiral excitation with four gradient cycles (diameter of 30 mm) with a flip angle of 60°. Temporal resolution of the navigator acquisition in each position was 82 ms. The patients were examined in the common supine position and no breathing commands were given. We used a gradient echo sequence scout to determine the position of the diaphragm, the thorax and the abdominal wall before placing the navigators. One navigator was placed through the dome of the right hemi-diaphragm to detect the cranial-caudal (CC) position of the diaphragm (Figure 1a). A second navigator was placed through the left hemi-diaphragm to also detect the CC position of diaphragm (Figure 1a). A third navigator was placed in anterior-posterior (AP) direction through the right chest wall at the height of the 3rd intercostal space to measure the AP position of the thorax (Figure 1b). A fourth navigator was positioned in left-right (LR) direction through the right chest wall at the height of the 3rd intercostal space to measure the LR position of the thorax (Figure 1c). A fifth navigator was positioned in AP direction through the abdominal wall at the height of the 5 cm below the xiphoid process to measure the AP position of the abdominal wall (Figure 1d). Since the available software only allowed the acquisition of three navigator echoes in one sequence two series with three navigators each were performed. These consisted of CC _right diaphragm_, CC _left diaphragm _and AP _thorax _and CC _right diaphragm_, LR _thorax _and AP _abdominal wall_. In each series data was acquired for one-minute. After the two navigator series acquisition, the phased array coil was removed from the patient. The 20-cm-wide tight-fitting BELT was wrapped around the patient's abdomen in expiration to suppress breathing related motion of the diaphragm (Figure 2) then the phased array coil was replaced on the patient's chest. A gradient echo sequence scout was then performed to position the navigators. Two further series of the navigator acquisition were performed using the same navigator positions as before the application of the BELT i.e. CC _right diaphragm_, CC _left diaphragm _and AP _thorax _and CC _right diaphragm_, LR _thorax _and AP _abdominal wall_.

After the scan, each patient completed the following brief questionnaire; how long would you tolerate the BELT if a future scan was needed? (A. 0 min, B. 0-10 min, B. 10-20 min, C. 20-30 min, D. > 30 min). We recorded the time of belt administration individually in each patient.

### Volunteer scanning

Non-contrast enhanced whole-heart coronary MRA was obtained in 6 normal volunteers (3 subjects in the UK and in Japan, respectively) with and without application of the BELT. Imaging was performed at 1.5-T (Achieva, Philips Medical Systems, Best, the Netherlands) using a 32-channel cardiac surface coil [16]. Drift correction was employed.

### Data Analysis

**Figure 3:**
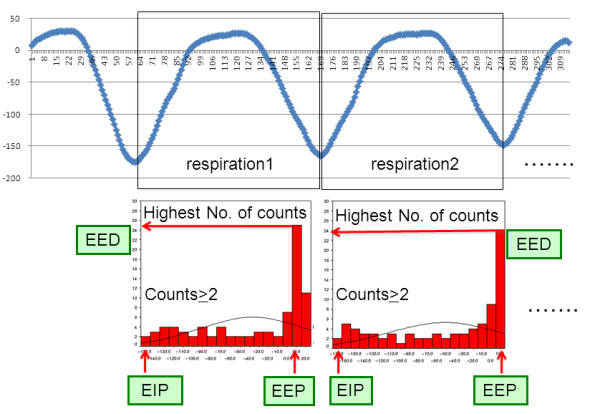
**Diaphragmatic position during 2 representative breathing cycles**. (a) Diaphragmatic position during 2 representative breathing cycles. (b) Corresponding histograms of the diaphragmatic position. The position with the highest number of counts was defined as EEP. The EED was defined as the number of counts within the EEP interval. The most distant position from EEP with at least 2 counts on the histogram was defined as the EIP.

The navigator data were exported from the console computer and were converted into PASW Statistics, version 18.0.2, software (SPSS, Chicago, Ill) for further processing. The positioning of diaphragm, thorax and abdominal wall was determined on the basis of the original navigator data. The diaphragmatic end expiratory position (EEP), end inspiratory position (EIP) and the duration of end expiration (EED) were calculated with histograms for steps of 1 mm on the basis of the diaphragmatic CC navigator positioned through the right diaphragm [15](Figure 3). The diaphragmatic position which occurred most frequently for each breathing cycle was defined as the EEP. The most distant position from EEP with at least 2 counts on the histogram was defined as the EIP. The EED was determined from the EEP values of the histogram for each breathing cycle. The EED was defined as the number of counts within the EEP interval. Each count corresponds to 82 ms. EEP-EIP was defined as the distance from EEP to EIP. Breathing cycle and respiration rate were calculated from the navigator data. Five-millimeter gating window was defined as the range between the lines of ± 2.5 mm up and down from the averaged EEP. The duration within the gating window in each respiration and that in one minute was calculated by counting the navigator position within the gating window. Scan efficiency (SE) was calculated as follows; SE = [the duration within 5 mm gating window per minutes]/[cardiac cycle]/[heart rate]. The different navigator echo signals were correlated with each other. The relationship between the different navigator signals was assessed by regression analysis. The slope and regression coefficient of this graph was obtained as a correction factor and a determinant of the relative movement respectively [15]. The correction factors for the thoracic AP and LR motion and abdominal AP motion in relation to right diaphragmatic CC motion after the application of the BELT were corrected by considering the decrease of EEP-EIP as follows; cCF = (100+%increase) × CF/100, where cCF is corrected correction factor, CF is corrected factor and %increase was defined as; (parameter _belt _- parameter _pre_)/parameter _pre _× 100.

Whole-heart coronary MRA images were analyzed using an image analysis workstation (Virtual Place, Aze, Tokyo, Japan). Curved multi-planar reconstruction (MPR) images were generated on the workstation.

### Statistical analysis

Statistical analysis was performed using PASW Statistics, version 18.0.2, software (SPSS, Chicago, Ill). For all continuous parameters, results are given as the mean ± standard deviation. For continuous variables, Shapiro-Wilk test was performed to see whether or not the variables were skewed. For continuous variables with normal distribution, parametric tests were applied. For discrete variables and skewed continuous variables, non-parametric statistics were applied. To detect statistically significant differences within a group, the paired Student *t *test or the Wilcoxon signed-rank test was used. Differences between two groups were tested by applying the unpaired Student *t *test or the Mann-Whitney *U *test. The correlation between age, sex, height, weight, BSA or abdominal circumference and differences in EEP-EIP, RR or Scan efficiency between before and after the BELT was evaluated. Pearson correlation or Spearman's rank correlation was used to test the relationship between two variables. All tests were two-tailed, and P < 0.05 was considered to indicate a statistically significant difference.

## Results

Height, weight, body surface area (BSA) and belly circumference in UK patients were significantly larger that that in Japanese patients (p = 0.007, p = 0.004, p = 0.038, p = 0.003, p = 0.016, respectively). The Japanese patients were significantly older than British patients (p < 0.001).

All patients accepted the application of the BELT. Acquisition of navigator position was completed in all patients (the BELT on time 15.2 ± 6.7 min). The belt on time did not differ between UK and Japanese patients (14.9 ± 5.3 min vs 15.3 ± 7.4, p = 0.318).

**Figure 4:**
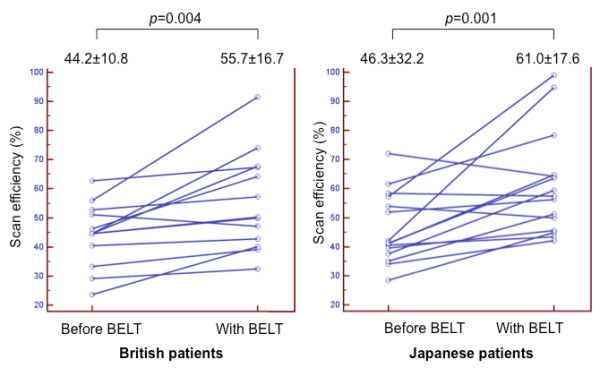
**Dots and line diagrams indicating the scan efficiency**. Dots and line diagrams indicating the scan efficiency in the UK patients (a) and Japanese patients (b). In both groups, scan efficiency was significantly increased after the application of the BELT.

EEP-EIP, EED, breathing cycle, RR, the duration within 5 mm gating window per minute, the duration within 5 mm gating window per respiration and scan efficiency before and after fitting the BELT are summarized in Table 2. After fitting the BELT, EEP-EIP decreased (all patients, 14.9 ± 6.2 mm to 9.4 ± 3.8 mm, p < 0.001; UK patients, 15.9 ± 6.0 mm to 9.7 ± 3.1 mm, p = 0.001; Japanese patients, 14.0 ± 6.4 mm to 9.1 ± 4.6 mm, p = 0.001), breathing cycle decreased (all patients, 6.71 ± 2.47 s to 5.83 ± 2.07 s, p = 0.002, UK patients, 6.71 ± 2.47 s to 5.83 ± 2.07 s, p = 0.002; Japanese patients, 6.48 ± 2.50 s to 5.50 ± 1.85 s, p = 0.041), RR increased (all patients, 10.0 ± 3.1 min^-1 ^to 11.2 ± 3.0 min^-1^, p = 0.003; UK patients, 9.5 ± 2.8 min^-1 ^to 10.7 ± 2.8 min^-1^, p = 0.038; Japanese patients, 10.4 ± 3.5 min^-1 ^to 11.8 ± 3.1 min^-1^, p = 0.036), the duration within 5 mm gating window per minute increased (all patients, 27.8 ± 7.8 s to 35.5 ± 1.8 s, p < 0.001; UK patients, 27.8 ± 8.5 s to 34.3 ± 9.6 s, p = 0.02; Japanese patients, 27.8 ± 7.3 s to 36.6 ± 10.6 s, p = 0.005) and the calculated scan efficiency using 5 mm gating window increased (all patients, 45.3 ± 11.4% to 58.6 ± 17.0%, p < 0.001; UK patients, 44.2 ± 10.8% to 55.7 ± 16.7%, p = 0.004; Japanese patients, 46.3 ± 32.2% to 61.0 ± 17.6%, p = 0.001, Figure 4). No significant differences were found in EED (all patients, 1.44 ± 0.78 s vs 1.42 ± 0.59 s, p = 0.905; UK patients, 1.49 ± 0.91 s vs 1.40 ± 0.65 s, p = 0.451; Japanese patients, 1.39 ± 0.67 s vs 1.44 ± 0.54 s, p = 0.41). The duration within 5 mm gating window per respiration was slightly increased but no significant difference was observed (all patients, 3.2 ± 1.5 s to 3.4 ± 1.4 s, p = 0.120; UK patients, 3.3 ± 1.7 s to 3.6 ± 1.9 s, p = 0.266; Japanese patients, 3.0 ± 1.3 s to 3.2 ± 0.9 s, p = 0.534, respectively). No significant differences were found between British and Japanese patients both before and after application of the BELT in the aforementioned parameters (Table 3). Moderate correlation was found between height and difference in EEP-EIP before and after the BELT (r_s _= 0.476, p = 0.008). There was no substantial correlation between other variables (Table 4).

**Table 2:** EEP-EIP, EED, breathing cycle, RR, the duration within 5 mm gating window per minute increased, the duration within 5 mm gating window per respiration and scan efficiency before and after fitting the BELT

		pre	BELT	*p value*
EIP-EEP (mm)	All	14.9 ± 6.2	9.4 ± 3.8	***< 0.001***
	
	UK	15.9 ± 6.0	9.7 ± 3.1	***0.001***
	
	Japan	14.0 ± 6.4	9.1 ± 4.6	***0.001***

EED (sec)	All	1.44 ± 0.78	1.42 ± 0.59	*0.905*
	
	UK	1.49 ± 0.91	1.40 ± 0.65	*0.451*
	
	Japan	1.39 ± 0.67	1.44 ± 0.54	*0.410*

Breathing cycle (sec)	All	6.71 ± 2.47	5.83 ± 2.07	***0.002***
	
	UK	6.93 ± 2.50	6.16 ± 2.28	***0.022***
	
	Japan	6.48 ± 2.50	5.50 ± 1.85	***0.041***

Respiratory rate (min^-1^)	All	10.0 ± 3.1	11.2 ± 3.0	***0.003***
	
	UK	9.5 ± 2.8	10.7 ± 2.8	***0.038***
	
	Japan	10.4 ± 3.5	11.8 ± 3.1	***0.036***

Duration within 5 mm gating window per respiration (sec)	All	3.2 ± 1.5	3.4 ± 1.4	*0.120*
	
	UK	3.3 ± 1.7	3.6 ± 1.9	*0.266*
	
	Japan	3.0 ± 1.3	3.2 ± 0.9	*0.534*

Duration within 5 mm gating window per minute (sec)	All	27.8 ± 7.8	35.5 ± 1.8	***< 0.001***
	
	UK	27.8 ± 8.5	34.3 ± 9.6	***0.020***
	
	Japan	27.8 ± 7.3	36.6 ± 10.6	***0.005***

Scan efficiency using 5 mm gating window (%)	All	45.3 ± 11.4	58.6 ± 17.0	***< 0.001***
	
	UK	44.2 ± 10.8	55.7 ± 16.7	***0.004***
	
	Japan	46.3 ± 32.2	61.0 ± 17.6	***0.001***

**Table 3:** Comparison between UK and Japanese patients in EEP-EIP, EED, breathing cycle, RR, the duration within 5 mm gating window per minute increased, the duration within 5 mm gating window per respiration and scan efficiency

		UK	Japan	p value
EIP-EEP (mm)	Pre	15.9 ± 6.0	14.0 ± 6.4	0.325

	BELT	9.7 ± 3.1	9.1 ± 4.6	0.250

EED (sec)	Pre	1.49 ± 0.91	1.39 ± 0.67	0.902

	BELT	1.40 ± 0.65	1.44 ± 0.54	0.806

Breathing cycle (sec)	Pre	6.93 ± 2.50	6.48 ± 2.50	0.512

	BELT	6.16 ± 2.28	5.50 ± 1.85	0.345

Respiratory rate (min^-1^)	Pre	9.5 ± 2.8	10.4 ± 3.5	0.427

	BELT	10.7 ± 2.8	11.8 ± 3.1	0.301

Duration within 5 mm gating window per respiration (sec)	Pre	3.3 ± 1.7	3.0 ± 1.3	0.605

	BELT	3.6 ± 1.9	3.2 ± 0.9	0.462

Duration within 5 mm gating window per minute (sec)	Pre	27.8 ± 8.5	27.8 ± 7.3	0.991

	BELT	34.3 ± 9.6	36.6 ± 10.6	0.683

Scan efficiency using 5 mm gating window (%)	Pre	44.2 ± 10.8	46.3 ± 32.2	0.621

	BELT	55.7 ± 16.7	61.0 ± 17.6	0.421

**Table 4:** The correlation between age, sex, height, weight, BMI, BSA, belly circumference and differences in EEP-EIP, RR or Scan efficiency between before and after the BELT

Differences between before and after the BELT		Age	Sex	Height	Weight	BSA	Belly circumference
Scan efficiency	r	0.052	0.189	0.231	0.116	0.164	-0.034
	
	*p*	0.795	0.334	0.237	0.558	0.404	0.865

EEP-EIP	r	-0.376	0.116	0.476	0.137	0.210	-0.025
	
	*p*	0.040	0.542	0.008	0.471	0.266	0.896

Respiratory rate	r	-0.093*	-0.268	-0.072*	-0.124	-0.138	-0.119
	
	*p*	0.625*	0.153	0.706*	0.515	0.468	0.531

*: Pearson correlation, no mark: Spearman's rank correlation

**Figure 5:**
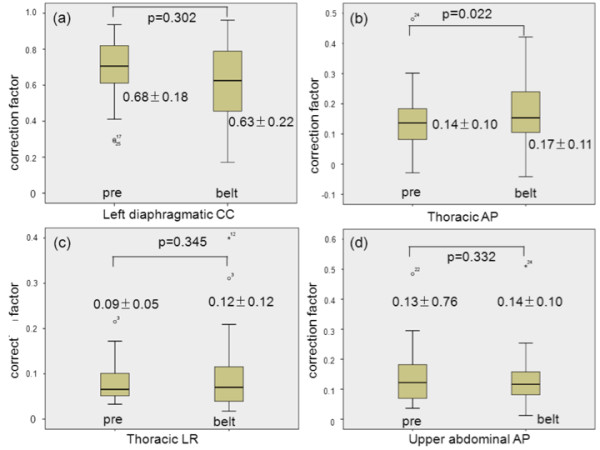
**Correction factors.** Correction factors for the left diaphragmatic CC motion (a), thoracic AP (b) and LR (c) motion and abdominal AP motion (d) in relation to right diaphragmatic CC motion after application of the BELT. Correction factors for thoracic AP (b) and LR (c) motion and abdominal AP motion (d) were corrected by considering the decrease of EEP-EIP, *see text*.

For all patients, correction factor of the right thoracic AP in relation to right diaphragmatic CC direction increased after fitting the BELT (0.14 ± 0.01 to 0.17 ± 0.11, p = 0.022), while no significant differences were found in correction factors of the left diaphragmatic CC, right thoracic LR and upper abdominal wall AP motion in relation to the right diaphragmatic CC motion (0.68 ± 0.18 to 0.63 ± 0.22, p = 0.302; 0.09 ± 0.05 to 0.12 ± 0.12, p = 0.345; 0.13 ± 0.76 to 0.14 ± 0.10, p = 332, respectively) (Figure 5). There were no significant differences in the regression coefficients between before and after the application of the BELT in each navigator position (Table 5).

**Table 5:** The regression coefficients of the relationship between right thoracic wall position in AP and LR direction and upper abdominal wall position in AP direction and right diaphragm position in CC direction before and after the application of the BELT

R^2^	pre	belt	*p value*
Left diaphragm position in CC direction versus right diaphragm position in CC direction.	0.88 ± 0.13	0.83 ± 0.15	0.088

Right thoracic wall position in AP direction versus right diaphragm position in CC direction	0.77 ± 0.22	0.73 ± 0.24	0.882

Right thoracic wall position in LR direction versus right diaphragm position in CC direction	0.71 ± 0.20	0.77 ± 0.12	0.552

upper abdominal wall in AP direction versus right diaphragm position in CC direction	0.80 ± 0.18	0.78 ± 0.25	0.955

**Figure 6:**
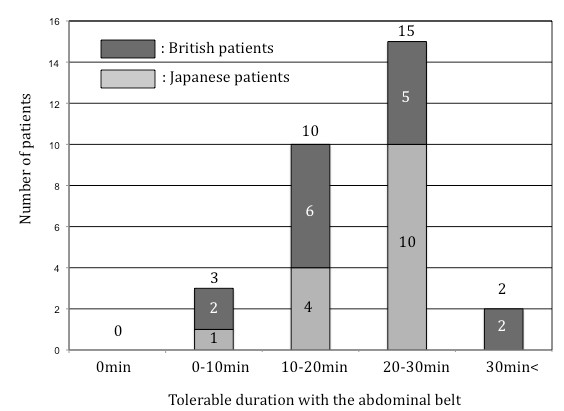
**Answers to the questionnaire**. Answers to the questionnaire (how long would you tolerate the BELT if a future scan was needed?) are summarized as bar graphs. No patients answered that they wouldn't tolerate the BELT. 90% of them would tolerate more than 10 minutes for future whole-heart scans.

The answers to the questionnaire are summarized in Figure 6. All patients answered that they would tolerate the BELT in a future examination. 90% of them would tolerate the BELT more than 10 minutes for future whole-heart scans.

**Figure 7:**
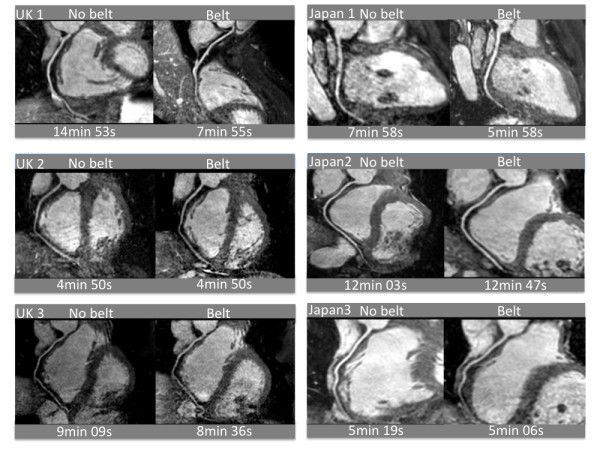
**Non-contrast enhanced whole-heart coronary MRA**. Non-contrast enhanced whole-heart coronary MRA were obtained in **6 **normal volunteers (3 subjects in the UK and in Japan, respectively) with and without application of the BELT. The individual scan durations are indicated below the images.

**Figure 8:**
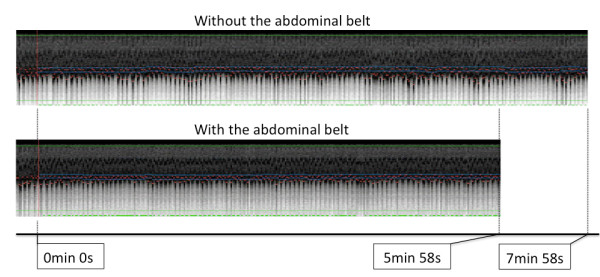
**The example of the navigator images**. The example of the navigator images obtained in one of the normal volunteers.

Curved MPR images obtained before and with the application of the BELT in each volunteer are displayed in Figure 7 along with the individual scan durations. An example of the navigator images obtained in one of the normal volunteers is shown in Figure 8.

## Discussion

The present study shows that the application of the BELT results in increased scan efficiency of navigator gated whole-heart coronary MRA both in UK and Japanese patients populations. This is mainly explained by the decrease of amplitude of diaphragmatic displacement, the unchanged end expiratory duration and increase of respiratory rate after fitting the BELT. In the current study, after the application of the BELT, EEP-EIP significantly decreased both in UK patients and Japanese patients, whereas EED remained almost unchanged in both groups. As a result of this, the duration of the diaphragm resting within the 5 mm gating window slightly increased in each breathing cycle. At the same time, the respiratory rate increased significantly in both groups. As a consequence of these factors, the duration of the diaphragm within the 5 mm gating window per minute significantly increased resulting in a significant increase in scan efficiency in both populations. These findings are probably the main mechanism by which the BELT reduces scan time and improves coronary image quality at the same time. Our findings differ from a previous report by Morita et al in 10 healthy volunteers who found no significant differences in acquisition time, navigator efficiency, and subjective image quality of whole-heart coronary MRA with and without an BELT [17]. These differences are most likely due to the fact that Morita et al. applied the belt in deep inspiration, whereas in our study the belt was applied in expiration. Our approach probably results in tighter compression of the abdomen and more restraint of diaphragmatic motion. The initial effect of the BELT was to restrict diaphragmatic motion. Tightening the belt further would theoretically lead to increased diaphragmatic restriction. However, it is important to tighten the BELT as much as possible without discomfort to the patient. Since the abdominal circumference tends to be largest during the deep inspiration, we speculated that the BELT would compress the abdomen most effectively during end-expiration.

The BELT restricts the amplitude of diaphragmatic motion. The same minute volume of ventilation is maintained by two different mechanisms namely an increase in respiratory rate and an increase in thoracic AP motion. No change in left-right motion of the thorax or AP motion of the upper abdomen was found.

In a standard WHCMRA scan, only one navigator is placed through the dome of the right hemi-diaphragm [4,10]. Correction of 0.6 of diaphragmatic CC displacement for cardiac CC position is widely used in the standard WHCMRA scans, which was determined by the relationship between motion of left main coronary artery and right diaphragm [18]. This approach relies on a simplified motion model assuming: 1) heart motion is linearly related to diaphragmatic motion; 2) a constant correction factor applies to all human subjects. In the current study, correction factor of the left diaphragmatic CC motion in relation to the right diaphragmatic CC motion was 0.68 before application of the BELT and 0.63 with the BELT. These values are very close to the standard correction factor for the CC heart motion (0.6) although a wide variation was observed among individuals. The relation (R^2^) between right diaphragmatic CC motion and left diaphragmatic CC motion, right thoracic AP or LR motion, and upper abdominal AP motion was between 0.7-0.9 and did not change after application of the belt.

No patients refused the BELT in the present study, and all patients stated that they would tolerate the BELT during a future examination. Further more, 90% of them would tolerate the BELT for more than 10 minutes. Application of the BELT is a well-tolerated physical intervention and can be routinely applied to whole-heart coronary MRA imaging.

### Limitations

Several study limitations should be acknowledged. Firstly, in this study, we only studied the navigator position rather than acquiring the actual whole-heart coronary MRA images. However we have demonstrated the feasibility of the BELT to improve image quality and scan duration in 3 respective MRA volunteer scans in the UK and in Japan. Secondly, navigator acquisition was done only for one minute. Due to these limitations, scan efficiency obtained in this study cannot be simply applied to a real WHCMRA scan. In future studies, whole-heart coronary MRA should be performed using the BELT technique employed in the present study. Scan duration, image quality, scan efficiency have to be compared before and after the administration of the BELT. Thirdly, direct influences of the BELT on cardiac motion were not evaluated in this study. Wide variation in the actual correction factor among individuals was reported indicating that usage of a fixed correlation factor will result in subject-dependent residual errors in heart position estimates [18]. Further investigation is required to evaluate if the BELT can reduce the variation of the correction factor among individuals.

## Conclusion

The results in the present study indicate that scan efficiency significantly increases using a BELT both in the UK and Japanese patients. This is mainly a result of a decrease in breathing amplitude in combination with longer end-expiratory resting times.

## Competing interests

Eike Nagel received major grant support from Philips Healthcare and Bayer Schering Pharma. The other authors declare that they have no competing interests.

## Authors' contributions

MI designed the study protocol, carried out the MR studies, analyzed the data and drafted the manuscript. AS participated in the study design, performed the MR studies and drafted the manuscript. ST, GM and AC helped to perform the MR studies and to draft the manuscript. BB helped with the statistical analysis. TS helped with parameter optimization. HS participated in the study design. EN designed the study protocol and drafted the manuscript. All authors read and approved the final manuscript.
